# Dislocation of a previously successful XEN glaucoma implant into the anterior chamber: a case report

**DOI:** 10.1186/s12886-017-0540-1

**Published:** 2017-08-22

**Authors:** Nikolaos Dervenis, Athanasia Maria Mikropoulou, Panagiotis Dervenis, Amanda Lewis

**Affiliations:** 1 Sussex Eye Hospital, Brighton, UK; 2 Department of Ophthalmology, University Hospital of Alexandroupolis, Alexandroupolis, Greece; 3 Aristotle University of Thessaloniki, Thessaloniki, Greece

**Keywords:** Migs, XEN implant, Glaucoma

## Abstract

**Background:**

XEN glaucoma implant (XEN gel stent, Aquesys, Inc) is a new minimally invasive device approved for the treatment of glaucoma.

**Case presentation:**

A 45 year old female was being followed and treated for primary open angle glaucoma in our tertiary referral center. Due to failure of medical treatment in controlling the glaucoma, surgery was offered to the patient. The XEN 45 μ-fistula implant was successfully placed in both eyes and adequate intraocular pressure control was achieved for 4 months. The left eye pressure then increased and the XEN implant was found in the anterior chamber. Topical intraocular pressure lowering therapy had to be re-initiated to achieve adequate pressure control.

**Conclusion:**

We describe a new potential complication of the XEN glaucoma implant.

## Background

Subconjunctival drainage of aqueous humor with bleb formation has been the most widely used surgical option for the treatment of glaucoma. Microinvasive glaucoma surgery (MIGS) has gained considerable interest during the last years and is expected to play an important role in the management of glaucoma patients in the future [1–3].

The XEN implant is one of the new commercially available MIGS devices that can be offered to glaucoma patients. It has recently been approved by the FDA for the treatment of glaucoma. The XEN Gel stent is a 6 mm hydrophilic tube with a 45-μm lumen size composed of a porcine collagen-derived gelatin crosslinked with glutaraldehyde. It has been designed to be non-degrading, cause no foreign body sensation, and bend and conform to tissue reducing risk of erosion. It comes preloaded and it can be injected ab interno creating a drainage pathway between the anterior chamber and the subconjunctival space. Subconjunctival injection of 0.2 mg/ml of mitomycin-C can be also used to improve the long-term outcomes. XEN implantation can be offered as a standalone procedure or in combination with cataract surgery and close postoperative follow up is recommended [1, 4].

Early reports about XEN implantation of different lumen tubes suggest favorable outcomes regarding the intraocular pressure control and number of pressure lowering medications [5, 6]. However despite the mechanical properties of the stent, cases of stent exposure have been described in the literature [7].

## Case presentation

In our report we present a case of dislocation of the XEN implant into the anterior chamber. Consent for the publication of this case report and any additional related information was taken from the patient involved in the study. A 45 year old female was being followed and treated for primary open angle glaucoma in our clinic. Pretreatment intraocular pressure was 31 mmHg in the right eye and 26 mmHg in the left eye and central corneal thickness was 496 μm and 494 μm respectively. The patient was asthmatic (treated with steroid inhalers) and was on immunosuppressive treatment (including short term courses of systemic steroids) because of severe eczema. She also presented with multiple allergies to pharmacological and non-pharmacological agents. Patient showed intolerance to topical preservatives so preservative free treatment had to be initiated. Despite maximum tolerated topical treatment with two IOP lowering medications (tafluprost 15 μm/ml once in the evening and dorzolamide 20 mg/ml preservative free twice per day) intraocular pressure was not well controlled in either of the eyes, being as high as 26 mmHg in both eyes. As the glaucoma was progressing and the pressure was not controlled, XEN implantation was offered to the patient for both eyes. After risks and benefits of surgery were discussed, patient consented to the treatment offered. XEN implantation was performed as a standalone procedure first in the left eye and then in the right eye on different dates. Since its introduction, XEN implant has gained its position in our unit in the management of open angle glaucoma and we have performed over 40 cases during the first year.

The routine surgical steps for XEN implantation in our unit followed in both eyes included: 0.2 mg/ml of Mitomycin C injected subconjunctivally at the area of planned XEN implantation, marking the conjunctiva 3 mm from the limbus superonasally, 7–0 silk as traction suture at the superior cornea, temporal clear cornea incision, filling of the anterior chamber with Healon GV (Abbott Medical Optics, Inc.) and use of the preloaded 27 gauge needle to insert the XEN implant at the superonasal part of the anterior chamber angle under gonioscopic view. Following that a 10–0 nylon suture was placed at the corneal incision and Healon GV was irrigated out of the anterior chamber. Following this technique only the tip of the stent projects in the anterior chamber and can be viewed during gonioscopy. The implant was successfully implanted in both eyes and patient received frequent topical steroids slowly tapering over the next 3 months and topical antibiotics for 1 week.

**Fig. 1:**
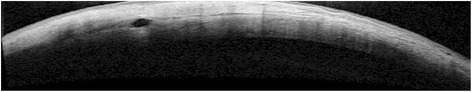
Anterior segment OCT showing the positioning of the implant in the sclera postoperatively

Surgery was successful in both eyes, with an un-eventful postoperative period and no adjunctive procedures (e.g. needling) were needed in either of the eyes. Slit lamp biomicroscopy and anterior segment OCT confirmed proper positioning of the XEN implant (Fig. 1). IOP was 14 mmHg in the right eye and 13 mmHg in the left eye on no drops 4 and 5 months after surgery respectively. Early cataract formation had been noted in the left eye.

**Fig. 2:**
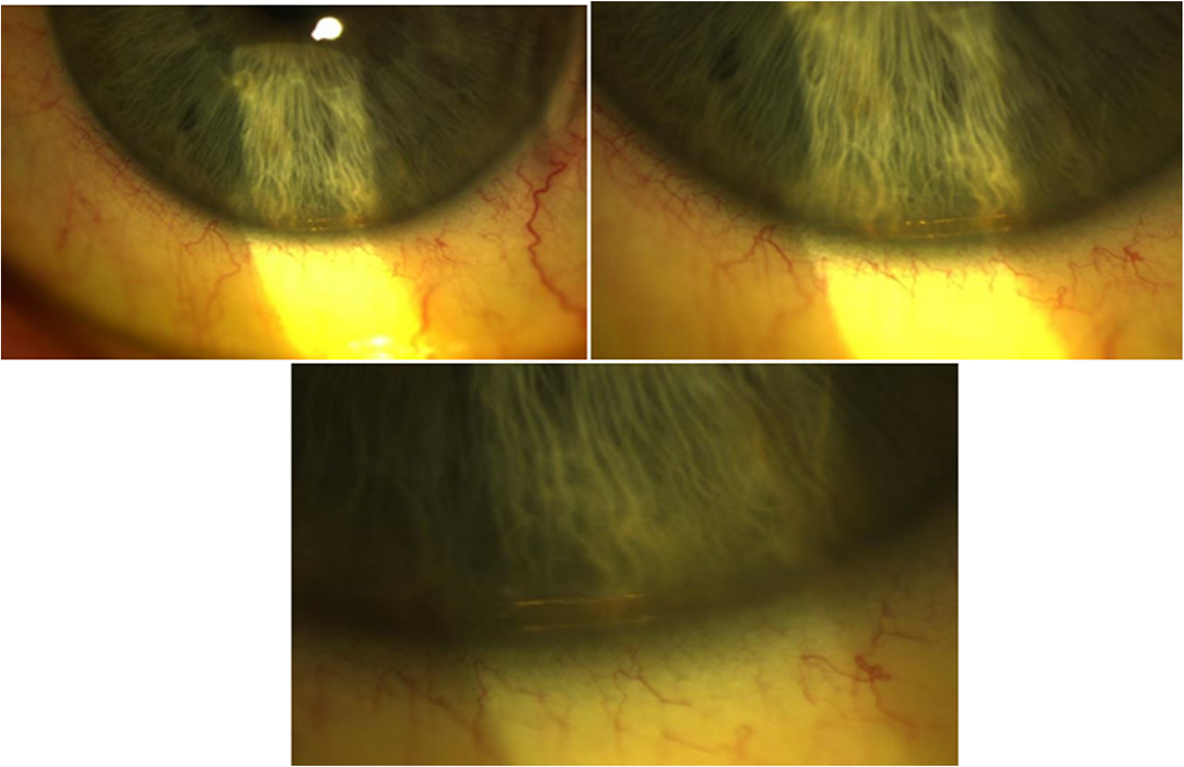
The dislocated XEN implant at the inferior anterior chamber angle

Six months after XEN implantation the patient presented to the Accidents and Emergency Department of our Eye Hospital complaining of a painful left eye for 1 week. Intraocular pressure was 15 mmHg in the left eye at that point and the anterior chamber was deep and quiet. Episcleral injection was noticed during the slit lamp biomicroscopy and the patient was diagnosed with diffuse unilateral episcleritis in the left eye and started with a 2 weeks tapering treatment of loteprednol etabonate (5 mg/ml). The patient was reviewed at the outpatients 1 month later. Episcleritis had resolved at that point but intraocular pressure was 35 mmHg and the whole XEN implant was noted to lie inferiorly in the anterior chamber angle (Fig. 2). The anterior chamber was quiet and there was no bleb at the area of previous XEN implantation. The patient had to start topical treatment again (tafluprost 15 μm/ml) in the left eye for adequate pressure control. The patient has since had left phacoemulsification and the XEN was removed from the anterior chamber at the same time.

## Discussion and conclusion

Our report suggests that dislocation of the XEN implant into the anterior chamber can happen after successful placement. The implant has been designed to become soft and swell up slightly within 1 or 2 min after implantation. This flexibility and tissue conforming of XEN has been suggested to reduce the risk of implant migration. More specifically the implant has been shown to form an “S” curve going through the scleral channel and this characteristic is supposed to mitigate potential migration. [1] However our repost suggests that the implant position is not fixed and secured and migration to the anterior chamber can happen. It is not clear whether the history of episcleritis had to do with the dislocation of the implant. Potentially inflammation of the episclera or external forces applied by the patient during the inflammation could have resulted to the implant dislocation. Therefore it is suggested that patients are advised to avoid rubbing the eye in case of XEN implantation. Other mechanisms (e.g. too wide scleral tunnel during implantation) cannot be excluded. However, improvements in the tube design could potentially increase the stability and reduce the risk of dislocation (e.g. progressive enlargement of the width of the tube from the anterior chamber to the subconjunctival space, anchored plate attached on the tube).

In conclusion, our report suggests a new potential complication of the placement of the XEN implant: dislocation to the anterior chamber. It is unclear if the episcleritis was a triggering factor and patients should be advised to avoid rubbing the eye after XEN placement. The implant was well tolerated in the anterior chamber and no anterior chamber inflammation was observed during the period of follow-up.

Financial Disclosure: Kestrel, who were marketing the product mentioned in the case study, paid for Miss Lewis to go on a training course to learn how to use the product. No disclosure for the rest of the authors

## Abbreviations

FDA: Food and Drug Administration
IOP: Intraocular pressure
MIGS: Microinvasive glaucoma surgery
OCT: Optical coherence tomography
